# Peak Total Bile Acids and Adverse Perinatal Outcomes in Intrahepatic Cholestasis of Pregnancy: A Retrospective Cohort Study with Threshold Analysis

**DOI:** 10.3390/jcm15207837

**Published:** 2026-10-11

**Authors:** Türkan Dikici Aktaş, Özgür Volkan Akbulut, Bengisu Elüstü Çakır, Emel Özalp, Belgin Savran Üçok, Şevki Çelen

**Affiliations:** 1 Department of Obstetrics and Gynecology, Ankara Etlik City Hospital, Ankara 06170, Türkiye; emelozalp@gmail.com (E.Ö.); drbelgin@gmail.com (B.S.Ü.); 2 Department of Perinatology, Ankara Etlik City Hospital, Ankara 06170, Türkiye; akbulutvolkan@yahoo.com (Ö.V.A.); bengisucakr@gmail.com (B.E.Ç.); sevkicelen@yahoo.com (Ş.Ç.)

**Keywords:** intrahepatic cholestasis of pregnancy, bile acids, perinatal outcome

## Abstract

**Background**: Peak total bile acids guide management of intrahepatic cholestasis of pregnancy (ICP), potentially influencing observed outcomes. We examined their association with a composite adverse perinatal outcome (CAPO) excluding preterm delivery. **Methods**: This retrospective cohort included 280 singleton pregnancies with ICP delivered at a tertiary hospital between January 2023 and May 2026. Peak bile acids were categorized as <40, 40–99, or ≥100 µmol/L. CAPO comprised meconium-stained amniotic fluid, nonreassuring fetal status, neonatal intensive care admission, respiratory distress syndrome, transient tachypnea, neonatal death, or intrauterine fetal death. Logistic regression and receiver operating characteristic analysis were performed; the exploratory threshold maximized the Youden index. **Results**: The categories included 185, 70, and 25 pregnancies, with CAPO in 31 (16.8%), 19 (27.1%), and 19 (76.0%), respectively (*p* < 0.001). In the ≥100 µmol/L group, the exact 95% confidence interval for CAPO was 54.9–90.6%. The apparent area under the curve was 0.700 (95% confidence interval 0.625–0.772). The 61.3 µmol/L threshold yielded 50.7% sensitivity and 83.9% specificity. Peak bile acids per 10 µmol/L (adjusted odds ratio 1.20, 95% confidence interval 1.11–1.31) and hypertensive disorders (3.27, 1.30–8.23) were independently associated with CAPO. **Conclusions**: Peak bile acids showed moderate discrimination within this managed cohort. The ≥100 µmol/L subgroup had higher morbidity but imprecise estimates. The same-cohort-derived 61.3 µmol/L threshold is exploratory and requires independent validation; it should not determine delivery timing.

## 1. Introduction

Intrahepatic cholestasis of pregnancy (ICP) is a pregnancy-specific liver disorder characterized by maternal pruritus, usually without a primary rash, and increased serum bile acid concentrations after competing causes of cholestasis have been excluded. Its frequency varies by ancestry, geography, season, and diagnostic practice. A recent global synthesis estimated a pooled incidence near 3%, while demonstrating substantial regional heterogeneity [1]. Maternal symptoms usually resolve after delivery, but fetal complications make the disorder clinically important [2]. Placental transfer and impaired fetal clearance of bile acids may expose the fetal myocardium, vasculature, and lungs to potentially toxic bile acid profiles [3].

Total serum bile acid concentration is the principal laboratory marker used for diagnosis and risk assessment. Diagnostic limits are not completely uniform because results vary with feeding status, gestational age, assay characteristics, and local reference ranges. The 2024 American Gastroenterological Association Clinical Practice Update defines ICP by pruritus with serum bile acids >10 µmol/L after exclusion of alternative causes, which corresponds to the diagnostic threshold used at our center [4]. Fasting samples may be more specific at low concentrations, but fasting testing can miss symptomatic patients whose randomly timed values are elevated [5]. These sources of variation complicate comparison of thresholds across cohorts.

Clinical management is mainly determined by the highest recorded bile acid concentration, gestational age, symptoms, coexisting pregnancy complications, and local guidance. The Society for Maternal-Fetal Medicine identifies ≥100 µmol/L as a high-risk level and recommends planned delivery at 36 weeks in these pregnancies [6]. The Royal College of Obstetricians and Gynaecologists classifies peak bile acids as mild at 19–39 µmol/L, moderate at 40–99 µmol/L, and severe at ≥100 µmol/L [7]. European guidance emphasizes repeated biochemical assessment and individualized delivery timing [8], and the Australian and New Zealand consensus similarly recognizes 40 and 100 µmol/L as clinically relevant severity boundaries [9].

The association between very high bile acid concentrations and stillbirth is well established, but stillbirth is rare and cannot be the sole outcome considered during routine counseling. Meconium-stained amniotic fluid, nonreassuring fetal status, neonatal respiratory morbidity, and neonatal intensive care unit admission occur more often and are clinically relevant. However, these outcomes are also influenced by surveillance and planned early delivery. A composite that includes delivery before 37 weeks can therefore become circular when the exposure itself determines delivery timing. Threshold performance in a retrospective managed cohort should be interpreted as discrimination within that management system, not as an estimate of untreated biological risk.

The primary objective was to evaluate the relationship between peak total bile acid concentration and CAPO excluding preterm delivery in a tertiary-center ICP cohort. Secondary objectives were to compare maternal, obstetric, and neonatal findings across the established <40, 40–99, and ≥100 µmol/L categories; identify variables independently associated with CAPO; and describe the apparent discriminatory performance of peak bile acids. Excluding preterm delivery did not make the remaining components independent of clinical management.

## 2. Materials and Methods

### 2.1. Study Design and Setting

We conducted a retrospective cohort study at the Department of Perinatology, Ankara Etlik City Hospital, a tertiary referral center in Ankara, Türkiye. Electronic records of patients followed for ICP between January 2023 and May 2026 were reviewed.

### 2.2. Participants

The cohort included pregnant individuals aged 18 years or older with a singleton pregnancy, clinically suspected ICP, an available total bile acid result, and delivery at the study hospital. Gestational age was established from the last menstrual period and confirmed by first-trimester ultrasonography when available. ICP was identified by pruritus without a primary rash together with total bile acids ≥ 10 µmol/L. Alternative hepatic and biliary diagnoses were assessed using the clinical history, available laboratory findings, and imaging when indicated. The exclusion criteria concerned documented alternative diagnoses and did not establish that every participant had undergone the same etiologic testing.

Exclusion criteria were multiple pregnancy, major fetal structural or chromosomal anomaly, fetal death before the diagnosis of ICP, preterm prelabor rupture of membranes, clinical chorioamnionitis, documented chronic liver disease, viral hepatitis, biliary obstruction, an acute gallstone episode, suspected drug-induced cholestasis, hemolysis with elevated liver enzymes and low platelets syndrome, acute fatty liver of pregnancy, severe preeclampsia with liver involvement, delivery at another hospital, unavailable total bile acid measurements, or critically incomplete neonatal records. The final analytic cohort comprised 280 pregnancies.

Individual autoimmune-serology results and postpartum hepatology assessments were unavailable for this analysis. Accordingly, exclusion of documented chronic liver disease should not be interpreted as confirmed universal screening for, or exclusion of, occult primary biliary cholangitis (PBC) or autoimmune hepatitis.

### 2.3. Exposure, Treatment, and Local Delivery Protocol

Total bile acid measurements obtained during routine care were abstracted as the first value, highest value, and value closest to delivery. The primary exposure was the peak total bile acid concentration because contemporary management is based on the highest documented level. Sampling was performed under routine-care conditions, and fasting status was not sufficiently standardized for a timing-specific analysis. Peak concentrations were categorized as <40, 40–99, and ≥100 µmol/L.

Management and delivery planning were based on the highest documented total bile acid concentration, gestational age, maternal symptoms, biochemical severity, coexisting maternal or fetal conditions, and fetal status. Ursodeoxycholic acid was prescribed at the discretion of the attending team according to symptoms, biochemical severity, gestational age, and the overall clinical context. During the study period, uncomplicated pregnancies with peak total bile acids < 40 µmol/L or 40–99 µmol/L were admitted at 37 + 0 weeks and delivery was planned during the 37th week. Pregnancies with peak total bile acids ≥ 100 µmol/L were generally admitted and scheduled for delivery at 36 + 0 weeks. Delivery before these planned gestational ages was undertaken for spontaneous labor, nonreassuring fetal status, or another maternal or fetal indication. Antenatal corticosteroids were administered for fetal lung maturation when birth before 37 weeks was anticipated, rather than as treatment for maternal cholestasis. The local plan for delivery at 36 + 0 weeks in the ≥100 µmol/L category provides a clinical explanation for the greater corticosteroid exposure in that group. Patient-level indication categories, administration-to-delivery intervals, and course-completion details were unavailable for additional reporting. The general indication for corticosteroids in anticipated preterm delivery is consistent with SMFM guidance [6].

UDCA exposure was summarized as use, daily dose in mg/day, and gestational age at initiation among treated patients. Individual dose–weight pairs, with maternal weight linked to the relevant dose and time point, were unavailable; therefore, weight-normalized doses in mg/kg/day could not be calculated. Treatment-attributed adverse-event data and a comprehensive profile of other concurrent medications were also unavailable. Absence of these data was not treated as absence of adverse events or medication exposure.

Aspartate aminotransferase, alanine aminotransferase, gamma-glutamyl transferase, total and direct bilirubin, albumin, platelet count, and international normalized ratio were recorded when available. Maternal variables included age, body mass index, gravidity, parity, smoking, mode of conception, previous cesarean delivery, gestational diabetes mellitus, hypertensive disorders, and fetal growth restriction or small-for-gestational-age status.

### 2.4. Outcomes

The primary outcome was CAPO, defined by the occurrence of at least one of the following: meconium-stained amniotic fluid; fetal distress or nonreassuring fetal status; neonatal intensive care unit (NICU) admission; respiratory distress syndrome; transient tachypnea of the newborn; neonatal death; or intrauterine fetal death. Delivery before 37 weeks was intentionally excluded because delivery timing was directly influenced by peak bile acids and the local protocol. Components were not mutually exclusive, and each pregnancy contributed once to CAPO regardless of the number of components present. Component descriptions and frequencies are provided in Supplementary Table S1. The composite was not severity-weighted and was not independent of clinical management.

Components are reported as recorded clinical events or diagnoses. Meconium-stained amniotic fluid denotes documented meconium in the amniotic fluid; fetal distress/nonreassuring fetal status denotes the recorded obstetric diagnosis; and NICU admission denotes a recorded neonatal intensive care admission. Respiratory distress syndrome and transient tachypnea of the newborn denote their respective recorded neonatal diagnoses and were not treated as interchangeable with any respiratory support. Intrauterine fetal death denotes death after ICP diagnosis and before birth, whereas neonatal death denotes a recorded death after live birth in the available neonatal outcome records. Detailed cardiotocographic, radiographic, and neonatal admission criteria were unavailable for standardized re-adjudication. The completeness of postdischarge mortality ascertainment could not be established. These record-based descriptions do not introduce additional diagnostic thresholds or imply a uniformly verified 28-day follow-up period.

### 2.5. Data Quality and Missing Data

Patient identifiers were replaced with study codes before analysis. Range, consistency, and logical checks were performed for gestational ages, bile acid categories, delivery timing, and outcome components. Umbilical artery pH was available for 216 neonates, and Apgar scores were available for 279. The observed minimum–maximum maternal age range and the additional clinical details described above were unavailable for reporting and could not be supplemented from the original charts during revision. These unavailable details were not imputed.

### 2.6. Statistical Analysis

Continuous variables were assessed with histograms, quantile plots, and the Shapiro–Wilk test. Approximately normally distributed variables are presented as mean ± standard deviation and were compared using Welch one-way analysis of variance because group sizes were unequal. Significant Welch tests were followed by Games-Howell comparisons. Skewed variables are presented as median (interquartile range) and were compared using the Kruskal–Wallis test, followed by pairwise Mann–Whitney U tests with Holm adjustment. Categorical variables are presented as number (percentage). Pearson chi-square testing was used when expected counts were adequate; the Fisher–Freeman–Halton exact test was used for sparse 3-by-2 tables. Significant categorical comparisons were followed by pairwise Fisher exact tests with Holm adjustment. The reported *p* values represent the omnibus three-group comparisons. For variables with a significant omnibus test, Holm-adjusted pairwise *p* values were calculated and reported separately. Because the Mann–Whitney U test evaluates the full rank distributions rather than the displayed medians alone, groups with identical or similar medians may still differ significantly. Adjusted *p* values were reported as <0.001 when below 0.001 and otherwise to three decimal places.

Univariable logistic regression evaluated associations with CAPO. Variables with univariable *p* < 0.20 were considered for multivariable analysis. The final model included five predictor terms: assisted conception, smoking, and hypertensive disorder as binary variables; gestational age at ICP diagnosis as a continuous variable per one-week increase; and peak total bile acids as a continuous variable per 10 µmol/L. Gestational age at diagnosis, rather than at delivery, was included. Gestational age at delivery was not included in the final model; it is influenced by disease severity and management and may lie on pathways to neonatal morbidity. UDCA use was examined in univariable analysis but did not meet the screening criterion (*p* = 0.383); dose and treatment-initiation timing were not included in the final model. Aspartate aminotransferase, alanine aminotransferase, and total bilirubin were not entered with peak bile acids because of collinearity. Antenatal corticosteroid exposure was excluded because it followed the decision to anticipate early delivery. Odds ratios are reported with 95% confidence intervals. This association model was not designed to estimate a causal treatment effect.

Receiver operating characteristic (ROC) analysis assessed the apparent discrimination of peak total bile acids for CAPO. The exploratory threshold was selected directly from the empirical ROC analysis by maximizing the Youden index (sensitivity + specificity − 1) in the full cohort; it was not a prespecified clinical threshold. A positive test was defined as a peak concentration at or above the evaluated threshold. Sensitivity, specificity, positive and negative likelihood ratios, and exact binomial 95% confidence intervals for sensitivity and specificity were reported at 40 µmol/L, the data-derived threshold, and 100 µmol/L. Exact two-sided Clopper–Pearson 95% confidence intervals were also calculated for selected outcome proportions from the reported event counts. The threshold was selected and evaluated in the same cohort; its performance estimates are apparent estimates, and the confidence intervals do not account for threshold-selection uncertainty. All tests were two-sided, and *p* < 0.05 indicated statistical significance. Analyses were performed with Python 3.12, NumPy 2.3.5, SciPy 1.17.0, statsmodels 0.14.6, and scikit-learn 1.8.0.

### 2.7. Ethics

The Ankara Etlik City Hospital No. 2 Scientific Research Evaluation and Ethics Committee approved the study (AEŞH-BADEK2-2026-508; 30 June 2026). The approved protocol involved anonymized retrospective records without additional testing, intervention, or patient contact. Data handling followed the Declaration of Helsinki and applicable institutional confidentiality requirements.

## 3. Results

### 3.1. Cohort Characteristics

The study included 280 singleton pregnancies with ICP. Peak total bile acids were <40 µmol/L in 185 patients (66.1%), 40–99 µmol/L in 70 (25.0%), and ≥100 µmol/L in 25 (8.9%). Mean maternal age was 31.0 ± 4.6 years, and mean body mass index was 27.7 ± 4.5 kg/m^2^. Maternal demographic and major obstetric characteristics did not differ significantly across bile acid categories. Gestational diabetes mellitus was recorded in 21 pregnancies (7.5%), hypertensive disorders in 23 (8.2%), and FGR or SGA in 16 (5.7%). These categories were not mutually exclusive. The observed minimum–maximum maternal age range was unavailable for reporting.

Gestational age at diagnosis decreased stepwise with increasing severity: 34.7, 33.9, and 31.9 weeks in the <40, 40–99, and ≥100 µmol/L groups, respectively (overall *p* < 0.001; all adjusted pairwise *p* < 0.001). First, peak, and predelivery bile acid concentrations also differed across every pairwise comparison. Aspartate and alanine aminotransferase concentrations were higher in the ≥100 µmol/L group than in either lower group, whereas total bilirubin increased across all three groups. Gamma-glutamyl transferase, albumin, and platelet count were similar (Table 1).

Ursodeoxycholic acid was used in 252 of 280 pregnancies (90.0%) and in every patient with peak total bile acids ≥ 100 µmol/L. Among users, median daily dose was 1000, 1500, and 1500 mg/day across the three severity groups, and treatment was initiated earlier as severity increased (Holm-adjusted pairwise *p* = 0.002 for <40 vs. 40–99 µmol/L and *p* < 0.001 for the other two comparisons). Antenatal corticosteroid exposure occurred in 49 pregnancies (17.5%) and was markedly higher in the ≥100 µmol/L group (80.0% vs. 10.8% and 12.9%; *p* < 0.001). Because dose–weight pairs and treatment-attributed adverse-event data were unavailable, no mg/kg/day comparison or adverse-event rate could be reported.

Exact adjusted pairwise *p* values (<40 vs. 40–99; <40 vs. ≥100; 40–99 vs. ≥100 µmol/L) were: gestational age at diagnosis, <0.001/<0.001/<0.001; first TBA, <0.001/<0.001/<0.001; peak TBA, <0.001/<0.001/<0.001; predelivery TBA, <0.001/<0.001/<0.001; AST, 0.936/<0.001/<0.001; ALT, 0.682/<0.001/<0.001; total bilirubin, <0.001/<0.001/<0.001; UDCA daily dose among users, <0.001/<0.001/0.001; gestational age at UDCA initiation, 0.002/<0.001/<0.001; and antenatal corticosteroid exposure, 0.661/<0.001/<0.001.

### 3.2. Obstetric and Neonatal Outcomes

Median gestational age at delivery was 37.0 weeks in both groups with peak total bile acids < 100 µmol/L and 36.0 weeks in the ≥100 µmol/L group (overall *p* < 0.001). All 25 pregnancies in the ≥100 µmol/L group delivered before 37 weeks, compared with 25 of 185 (13.5%) in the <40 µmol/L group and 13 of 70 (18.6%) in the 40–99 µmol/L group. This pattern was driven mainly by iatrogenic preterm delivery, which occurred in 84.0% of the severe group; spontaneous preterm delivery did not differ significantly. Labor induction increased with severity, while cesarean delivery rates were similar. In adjusted pairwise analyses, gestational age at delivery differed between the <40 and 40–99 µmol/L groups (Holm-adjusted *p* = 0.033), while both comparisons involving the ≥100 µmol/L group were *p* < 0.001. The diagnosis-to-delivery interval was longer in both higher-bile-acid groups than in the <40 µmol/L group, with no significant difference between the 40–99 and ≥100 µmol/L groups. Preterm delivery < 37 weeks and iatrogenic preterm delivery were significantly more frequent in the ≥100 µmol/L group than in either lower group; the two lower groups did not differ significantly.

Meconium-stained amniotic fluid occurred in 4.3%, 15.7%, and 32.0% of the <40, 40–99, and ≥100 µmol/L groups, respectively (*p* < 0.001). The <40 group differed from both higher groups in adjusted pairwise testing, whereas the 40–99 and ≥100 groups did not differ significantly. Birth weight was lower in the ≥100 µmol/L group than in the <40 µmol/L group. Median umbilical artery pH was lower in the ≥100 µmol/L group than in each lower group.

NICU admission occurred in 8.6%, 7.1%, and 48.0% of the three groups, respectively (*p* < 0.001). All four cases of respiratory distress syndrome occurred in the ≥100 µmol/L group. Any respiratory support was required in 3.2%, 0%, and 36.0%, respectively (*p* < 0.001). Neonatal hypoglycemia and phototherapy were also more frequent in the highest category. There were no recorded neonatal deaths. One intrauterine fetal death occurred in the ≥100 µmol/L group (1/25, 4.0%; exact 95% CI 0.1–20.4%). In that group, NICU admission was 12/25 (48.0%; 95% CI 27.8–68.7%) and respiratory distress syndrome was 4/25 (16.0%; 95% CI 4.5–36.1%) (Table 2). These are pregnancy-based proportions, including the pregnancy ending in fetal death in the denominator, to maintain consistency with the pregnancy-level composite. Adjusted pairwise testing showed that NICU admission, respiratory distress syndrome, and any respiratory support were significantly more frequent in the ≥100 µmol/L group than in either lower group. For neonatal hypoglycemia and phototherapy, the significant difference was confined to the <40 versus ≥100 µmol/L comparison.

Exact adjusted pairwise *p* values (<40 vs. 40–99; <40 vs. ≥100; 40–99 vs. ≥100 µmol/L) were: gestational age at delivery, 0.033/<0.001/<0.001; diagnosis-to-delivery interval, 0.003/0.001/0.214; preterm delivery < 37 weeks, 0.328/<0.001/<0.001; iatrogenic preterm delivery, 0.158/<0.001/<0.001; labor induction, 0.039/0.154/1.000; meconium-stained amniotic fluid, 0.011/<0.001/0.090; birth weight (Games-Howell), 0.284/0.006/0.112; 5 min Apgar score, 0.253/0.008/0.270; umbilical artery pH, 0.835/0.019/0.034; NICU admission, 0.803/<0.001/<0.001; respiratory distress syndrome, 1.000/<0.001/0.008; neonatal hypoglycemia, 1.000/0.032/0.055; phototherapy, 0.204/0.022/0.234; and CAPO, 0.077/<0.001/<0.001.

### 3.3. Composite Outcome and Threshold Analysis

CAPO occurred in 69 pregnancies (24.6%; exact 95% CI 19.7–30.1%). Frequencies were 31/185 (16.8%; 95% CI 11.7–22.9%) in the <40 µmol/L group, 19/70 (27.1%; 95% CI 17.2–39.1%) in the 40–99 µmol/L group, and 19/25 (76.0%; 95% CI 54.9–90.6%) in the ≥100 µmol/L group (*p* < 0.001). The two lower groups did not differ significantly after multiplicity adjustment, whereas the ≥100 µmol/L group differed from each lower group. Pregnancies with CAPO were diagnosed earlier than those without [33.4 (32.1–34.9) vs. 34.4 (33.4–35.4) weeks; *p* < 0.001] and had higher first bile acids [39.0 (19.8–70.2) vs. 21.4 (15.5–34.0) µmol/L], peak bile acids [61.3 (28.5–116.7) vs. 31.1 (22.0–50.5) µmol/L], and predelivery bile acids [34.2 (19.8–72.1) vs. 20.0 (14.1–32.6) µmol/L] (all *p* < 0.001).

**Figure 1:**
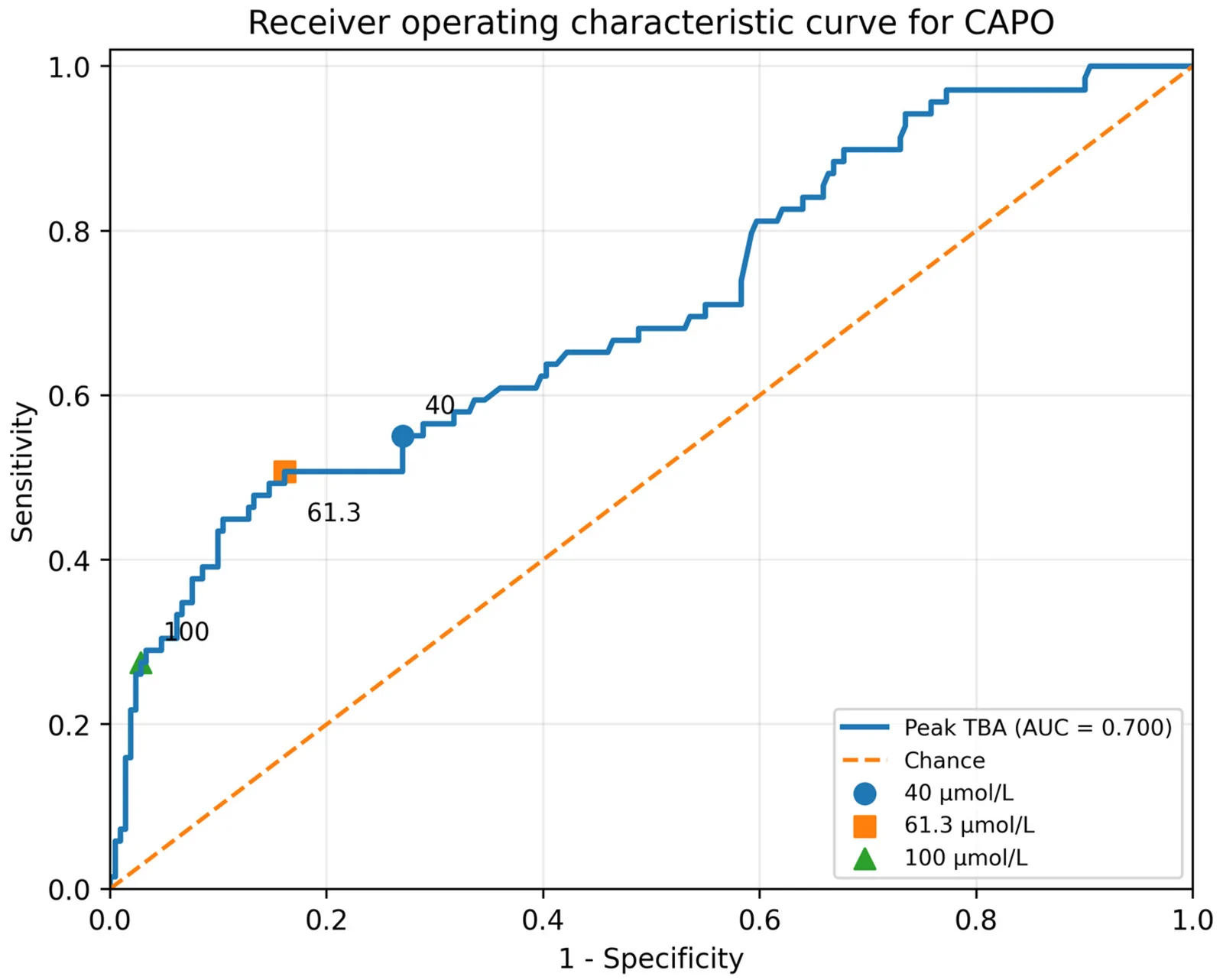
Receiver operating characteristic curve of peak total bile acids for the composite adverse perinatal outcome.

Peak total bile acids had an apparent AUC of 0.700 (95% confidence interval 0.625–0.772; *p* < 0.001). At 40 µmol/L, sensitivity was 55.1% and specificity was 73.0%. Maximizing the Youden index in the same cohort identified 61.3 µmol/L (Youden J = 0.346), with 50.7% sensitivity and 83.9% specificity. This ROC-derived value coincided with the reported median peak concentration in the CAPO group but was selected using the Youden criterion, not the group median. At 100 µmol/L, sensitivity was 27.5% and specificity was 97.2% (Table 3 and Figure 1).

### 3.4. Univariable and Multivariable Analyses

In univariable analysis, assisted conception, hypertensive disorder, earlier diagnosis, higher peak bile acids, higher aminotransferase concentrations, higher total bilirubin, and antenatal corticosteroid exposure were associated with CAPO at *p* < 0.05; smoking met the prespecified *p* < 0.20 screening criterion. In the final multivariable model, peak total bile acids remained independently associated with CAPO (adjusted odds ratio 1.20 per 10 µmol/L, 95% confidence interval 1.11–1.31; *p* < 0.001), as did hypertensive disorder (adjusted odds ratio 3.27, 95% confidence interval 1.30–8.23; *p* = 0.012). The estimate for assisted conception was imprecise and did not reach conventional statistical significance (adjusted odds ratio 4.05, 95% confidence interval 0.88–18.69; *p* = 0.073) (Table 4).

## 4. Discussion

This retrospective cohort demonstrated a clear severity gradient between peak total bile acid concentration and clinically important perinatal morbidity after preterm delivery was removed from the composite endpoint. CAPO increased from 16.8% below 40 µmol/L to 27.1% at 40–99 µmol/L and 76.0% at ≥100 µmol/L. Peak bile acids provided moderate discrimination, with an apparent AUC of 0.700. The 61.3 µmol/L threshold favored specificity over sensitivity. These findings support peak bile acids as a risk marker but do not support a single local cut-off as an independent delivery rule.

The strongest external evidence for biochemical risk stratification comes from the individual participant data meta-analysis by Ovadia et al., in which the stillbirth signal in singleton pregnancies was concentrated at peak bile acids ≥ 100 µmol/L [10]. Earlier work identified 40 µmol/L as a clinically relevant boundary for fetal complications [11], and a prospective population-based study reported higher risks of preterm delivery, neonatal unit admission, meconium-stained fluid, and stillbirth in severe ICP [12]. Our cohort was not powered to estimate stillbirth risk, but the marked increase in nonfatal morbidity at ≥100 µmol/L was directionally consistent with this severity framework.

Brouwers et al. reported increasing spontaneous preterm birth, meconium-stained amniotic fluid, and perinatal death with rising bile acid concentrations in a multicenter Dutch cohort [13]. Our findings were consistent with the graded increase in meconium and neonatal unit use, although our local delivery protocol produced a predominantly iatrogenic preterm pattern in the severe group. Kawakita et al. found that bile acids ≥ 40 µmol/L were independently associated with meconium-stained fluid, but no measured predictor remained associated with their adjusted composite neonatal morbidity [14]. This distinction is important: individual outcomes and broad composites do not necessarily share the same predictors. Other cohorts have also linked maximum bile acids >40 µmol/L with meconium, neonatal unit admission, and neonatal morbidity [15].

The data-derived threshold of 61.3 µmol/L was higher than the conventional 40 µmol/L boundary. Its specificity was 83.9%, but sensitivity was only 50.7%. A Turkish study reported a lower threshold near 34 µmol/L for preterm birth and neonatal unit admission [16], illustrating how the selected endpoint and local management can move an apparent optimum. Sarker et al. described progressively higher risks across several bile acid bands rather than a single abrupt transition [17], and a 2024 study of 15,826 patients similarly found increasing risks across a broad range of outcomes [18]. A recent Chinese cohort also demonstrated graded increases in indicated preterm birth, meconium, and neonatal admission with higher bile acids [19]. Taken together, these data favor tiered risk assessment over a universal binary cut-off. The threshold was optimized and evaluated using the same observations and may therefore be overfitted to this sample. The reported sensitivity and specificity are not independently validated operating characteristics, and their confidence intervals do not incorporate threshold-selection uncertainty. The threshold should not be used to define a low-risk group or trigger delivery without external evaluation.

Removing preterm delivery from CAPO avoided directly counting an outcome that was frequently determined by the bile-acid-based delivery protocol, but it did not eliminate management-related bias. More intensive fetal surveillance may increase the detection and recording of nonreassuring fetal status. Planned delivery timing changes gestational age at birth and may alter respiratory morbidity and NICU admission, while induction and mode of delivery may also affect these outcomes. NICU admission additionally depends on local observation practices, admission thresholds, and clinical judgment. Conversely, timely intervention may prevent fetal deterioration or death. Thus, clinical management may influence different components in different directions. The observed association and ROC curve describe outcomes under the local management strategy, not the untreated biological effect of bile acids or a management-independent endpoint.

Multivariable analysis provided additional context. Peak bile acids remained independently associated with CAPO after adjustment for assisted conception, smoking, hypertensive disorder, and gestational age at diagnosis. Hypertensive disorders also remained independently associated with CAPO, supporting integration of comorbidity into counseling rather than reliance on bile acids alone. Aminotransferases and bilirubin were associated with CAPO in univariable analyses but were not entered with peak bile acids because they were correlated biochemical markers. Antenatal corticosteroid exposure was deliberately excluded from the adjusted model because it was a downstream consequence of anticipated early delivery. Adjustment for gestational age at diagnosis should not be equated with adjustment for gestational age at delivery. UDCA use was considered but not retained under the univariable screening rule; this does not demonstrate an absence of treatment influence. Because 90% of the cohort and all patients in the highest category received UDCA, comparisons are vulnerable to confounding by indication and limited treatment overlap. Residual effects of treatment and delivery management remain possible.

Peak rather than first bile acid concentration was used because ICP severity can progress during gestation. A 2025 analysis reported that approximately one quarter of patients with repeated measurements moved to a higher severity category, which could alter delivery planning [20]. In our cohort, the severe group was diagnosed earlier and had a longer diagnosis-to-delivery interval, providing more opportunity for serial testing and for the observed peak to rise. This supports repeated bile acid measurement when symptoms persist or the clinical condition changes.

Meconium-stained fluid showed a strong stepwise pattern, whereas fetal distress did not reach conventional statistical significance. Individual outcomes have different mechanisms and different susceptibility to surveillance and delivery timing. Population-based data have shown increased preterm delivery and fetal complications in ICP, but absolute risks vary by population and treatment era [21]. Respiratory distress syndrome occurred only in the ≥100 µmol/L group. Prior neonatal work linked maternal ICP to respiratory distress and proposed bile-acid-related surfactant dysfunction as a possible mechanism [22]. The small number of respiratory distress syndrome events in our cohort requires cautious interpretation. Across the entire cohort, NICU admission (33/280, 11.8%), meconium-stained fluid (27/280, 9.6%), and nonreassuring fetal status (24/280, 8.6%) were the most frequent components. Respiratory distress syndrome (4/280, 1.4%), transient tachypnea (6/280, 2.1%), and fetal death (1/280, 0.4%) were less frequent, and no neonatal deaths were recorded. Because components overlap, these marginal frequencies cannot identify how many composite-positive pregnancies were attributable exclusively to any one component. The composite signal should therefore not be interpreted as a mortality signal.

Ursodeoxycholic acid was used in 90% of the cohort and in all patients with peak bile acids ≥ 100 µmol/L. The PITCHES randomized trial did not reduce its primary composite perinatal outcome [23]. Serum bile acids decreased in both treatment groups, but the adjusted comparison showed less reduction with ursodeoxycholic acid than with placebo (adjusted geometric mean ratio 1.18, *p* = 0.030), while alanine aminotransferase decreased. In the subsequent individual participant data meta-analysis, ursodeoxycholic acid was associated with a significant reduction in total preterm birth in randomized trials (adjusted odds ratio 0.61, 95% confidence interval 0.40–0.92), but a clear stillbirth benefit was not established [24]. Treatment in our cohort was not randomized and was strongly linked to severity; therefore, we did not estimate a treatment effect. The available daily dose summaries cannot establish equivalent exposure in mg/kg/day. Treatment-attributed adverse events could not be assessed, and no conclusion about UDCA safety can be drawn from their absence in the reported data.

Genetic susceptibility may also contribute to heterogeneity within clinically diagnosed ICP. Dixon et al. identified heterozygous, potentially damaging variants in ABCB4, ABCB11, ATP8B1, ABCC2, and TJP2 among 147 women with ICP [25]. These findings connect pregnancy-associated cholestasis with genes implicated in inherited cholestatic disorders, but do not mean that variant carriers necessarily have progressive familial intrahepatic cholestasis. Genotype-specific associations were not evaluated in the present analysis, so genetic susceptibility cannot be separated from other determinants of biochemical severity or outcomes.

Underlying cholestatic or autoimmune liver disease is another potential source of heterogeneity. PBC may be clinically subtle, and pregnancy-associated pruritus can reveal or aggravate pre-existing cholestatic disease [8,26]. Excluding documented chronic liver disease is not equivalent to excluding occult PBC or autoimmune hepatitis in every participant. Appropriate etiologic assessment may include antimitochondrial antibodies and PBC-specific antinuclear antibodies, with hepatology evaluation and reassessment of persistent postpartum biochemical abnormalities [8,26]. Individual autoimmune-serology results and postpartum assessments were unavailable in this study; universal negative testing therefore cannot be assumed. Unrecognized underlying disease may have contributed to diagnostic heterogeneity, and this limitation should be considered when interpreting the associations.

This study has limitations. Retrospective records are vulnerable to documentation error and residual confounding. Sampling time and fasting status were not standardized, and measurement frequency probably differed by severity. Only 25 pregnancies were in the ≥100 µmol/L category: the 76.0% CAPO proportion had a wide exact 95% CI of 54.9–90.6%, and the single fetal death corresponded to 4.0% with a 95% CI of 0.1–20.4%. These data do not support a precise stillbirth-risk estimate or reliable component-specific adjusted models for rare events. The heterogeneous composite included overlapping outcomes and remained sensitive to surveillance, delivery timing, and neonatal admission practices. Record-based component diagnoses could not be re-adjudicated against uniform detailed criteria, and complete postdischarge mortality ascertainment could not be confirmed. Umbilical artery blood gas results were missing for 64 neonates.

The observed maternal age range, paired UDCA dose and weight measurements, treatment-attributed adverse events, a comprehensive concurrent-medication profile, and individual autoimmune-serology results were unavailable. These omissions limit description of the cohort, evaluation of treatment exposure and safety, and exclusion of occult autoimmune liver disease. Genetic susceptibility was not evaluated. The endpoint was refined during the analytic revision, and both the cut-off and regression model were developed in the same single-center cohort. Univariable screening may also have omitted clinically relevant confounders. The reported discrimination is apparent rather than independently validated; external validation is required before the selected threshold is considered for clinical use.

## 5. Conclusions

Peak total bile acid concentration was associated with adverse perinatal morbidity among singleton pregnancies with ICP managed under the local clinical protocol. The highest observed CAPO frequency occurred at ≥100 µmol/L (19/25, 76.0%; exact 95% CI 54.9–90.6%). This pattern is consistent with the established severity framework, but the small subgroup and management-sensitive outcomes limit precision and causal interpretation.

Peak bile acids showed moderate overall discrimination. The center-specific value of 61.3 µmol/L provided greater specificity than sensitivity and cannot safely exclude risk below that level. It should not replace the established 40 and 100 µmol/L categories or determine delivery timing in isolation.

Clinical decisions should integrate the highest measured bile acid concentration with gestational age, symptom course, hypertensive and other pregnancy comorbidities, fetal assessment, and the risks of iatrogenic prematurity. Prospective multicenter studies should standardize sampling, separate management-related outcomes from disease-related outcomes, and validate prediction models in independent populations.

## Figures and Tables

**Table 1:** Maternal and laboratory characteristics according to peak total bile acid category.

Variable	<40 µmol/L (*n* = 185)	40–99 µmol/L (*n* = 70)	≥100 µmol/L (*n* = 25)	Overall *p* Value
Maternal age, years	31.1 ± 4.5	30.8 ± 4.8	30.6 ± 4.9	0.850
Body mass index, kg/m^2^	27.8 ± 4.6	27.0 ± 4.3	29.1 ± 4.0	0.103
Nulliparity	82 (44.3)	27 (38.6)	7 (28.0)	0.255
Assisted conception	6 (3.2)	1 (1.4)	2 (8.0)	0.233
Smoking	10 (5.4)	3 (4.3)	3 (12.0)	0.320
Previous cesarean delivery	38 (20.5)	13 (18.6)	5 (20.0)	0.940
Gestational diabetes mellitus	15 (8.1)	4 (5.7)	2 (8.0)	0.768
Hypertensive disorder	15 (8.1)	6 (8.6)	2 (8.0)	1.000
FGR or SGA	9 (4.9)	5 (7.1)	2 (8.0)	0.603
Gestational age at diagnosis, weeks	34.7 (33.6–35.4) ^a^	33.9 (32.5–34.9) ^b^	31.9 (31.3–33.4) ^c^	<0.001
First TBA, µmol/L	18.9 (14.3–22.6) ^a^	45.0 (39.7–56.6) ^b^	106.5 (78.5–117.2) ^c^	<0.001
Peak TBA, µmol/L	27.0 (20.7–32.3) ^a^	65.3 (55.4–77.8) ^b^	141.4 (124.5–160.0) ^c^	<0.001
Predelivery TBA, µmol/L	18.2 (13.6–22.4) ^a^	43.1 (34.8–55.5) ^b^	96.5 (81.3–112.8) ^c^	<0.001
Aspartate aminotransferase, U/L	29 (21–36) ^a^	30 (22–38) ^a^	48 (37–57) ^b^	<0.001
Alanine aminotransferase, U/L	34 (23–53) ^a^	34 (26–45) ^a^	60 (43–81) ^b^	<0.001
Gamma-glutamyl transferase, U/L	21 (16–27)	21 (16–30)	24 (19–28)	0.188
Total bilirubin, mg/dL	0.53 (0.43–0.65) ^a^	0.68 (0.57–0.84) ^b^	1.05 (0.87–1.15) ^c^	<0.001
Albumin, g/dL	3.32 ± 0.28	3.25 ± 0.30	3.22 ± 0.33	0.098
Platelet count, ×10^3^/µL	228.4 ± 45.5	233.9 ± 47.5	227.6 ± 39.6	0.684
Ursodeoxycholic acid use	163 (88.1)	64 (91.4)	25 (100.0)	0.161
UDCA daily dose among users, mg/day	1000 (750–1000) ^a^	1500 (1000–1500) ^b^	1500 (1000–2000) ^c^	<0.001
Gestational age at UDCA initiation among users, weeks	34.9 (33.7–35.6) ^a^	34.1 (32.8–35.1) ^b^	32.0 (31.4–33.7) ^c^	<0.001
Antenatal corticosteroid exposure	20 (10.8) ^a^	9 (12.9) ^a^	20 (80.0) ^b^	<0.001

Data are mean ± standard deviation, median (interquartile range), or *n* (%). Normally distributed variables were compared using Welch one-way analysis of variance with Games-Howell post hoc testing; skewed variables used Kruskal–Wallis with Holm-adjusted Mann–Whitney U tests; categorical variables used Pearson chi-square or the Fisher–Freeman–Halton exact test with Holm-adjusted pairwise Fisher tests. Different superscript letters indicate significant pairwise differences at adjusted *p* < 0.05; values sharing a letter did not differ significantly. UDCA dose and initiation timing were analyzed among users (*n* = 163, 64, and 25). FGR, fetal growth restriction; SGA, small for gestational age; TBA, total bile acid; UDCA, ursodeoxycholic acid. Pairwise post hoc results were as follows: gestational age at diagnosis, first TBA, peak TBA, predelivery TBA, total bilirubin, UDCA daily dose, and gestational age at UDCA initiation differed significantly across all three pairwise comparisons. For AST, ALT, and antenatal corticosteroid exposure, the ≥100 µmol/L group differed significantly from both the <40 and 40–99 µmol/L groups, whereas the two lower groups did not differ significantly. UDCA doses are recorded daily amounts in mg/day; individual mg/kg/day doses and treatment-attributed adverse-event rates were unavailable.

**Table 2:** Obstetric and neonatal outcomes according to peak total bile acid category.

Outcome	<40 µmol/L (*n* = 185)	40–99 µmol/L (*n* = 70)	≥100 µmol/L (*n* = 25)	Overall *p* Value
Gestational age at delivery, weeks	37.0 (37.0–37.1) ^a^	37.0 (37.0–37.1) ^b^	36.0 (35.6–36.3) ^c^	<0.001
Diagnosis-to-delivery interval, days	16 (11–23) ^a^	21 (13–30) ^b^	27 (15–30) ^b^	<0.001
Preterm delivery < 37 weeks	25 (13.5) ^a^	13 (18.6) ^a^	25 (100.0) ^b^	<0.001
Spontaneous preterm delivery	10 (5.4)	3 (4.3)	4 (16.0)	0.111
Iatrogenic preterm delivery	15 (8.1) ^a^	10 (14.3) ^a^	21 (84.0) ^b^	<0.001
Labor induction	112 (60.5) ^a^	54 (77.1) ^b^	20 (80.0) ^ab^	0.014
Cesarean delivery	82 (44.3)	28 (40.0)	10 (40.0)	0.787
Meconium-stained amniotic fluid	8 (4.3) ^a^	11 (15.7) ^b^	8 (32.0) ^b^	<0.001
Fetal distress/nonreassuring status	12 (6.5)	7 (10.0)	5 (20.0)	0.076
Birth weight, g	2810 ± 305 ^a^	2738 ± 345 ^ab^	2583 ± 318 ^b^	0.004
1 min Apgar score	9 (8–9)	9 (8–9)	9 (8–9)	0.051
5 min Apgar score	10 (10–10) ^a^	10 (10–10) ^ab^	10 (10–10) ^b^	0.020
Umbilical artery pH	7.264 (7.239–7.290) ^a^	7.261 (7.243–7.280) ^a^	7.217 (7.200–7.264) ^b^	0.023
NICU admission	16 (8.6) ^a^	5 (7.1) ^a^	12 (48.0) ^b^	<0.001
NICU stay among admitted neonates, days	2.5 (2.0–4.0)	3.0 (1.0–4.0)	3.0 (3.0–4.2)	0.182
Respiratory distress syndrome	0 (0.0) ^a^	0 (0.0) ^a^	4 (16.0) ^b^	<0.001
Transient tachypnea of the newborn	5 (2.7)	0 (0.0)	1 (4.0)	0.232
Any respiratory support	6 (3.2) ^a^	0 (0.0) ^a^	9 (36.0) ^b^	<0.001
Neonatal hypoglycemia	8 (4.3) ^a^	3 (4.3) ^ab^	5 (20.0) ^b^	0.017
Phototherapy	15 (8.1) ^a^	11 (15.7) ^ab^	7 (28.0) ^b^	0.009
Suspected neonatal sepsis	1 (0.5)	2 (2.9)	1 (4.0)	0.135
Neonatal death	0 (0.0)	0 (0.0)	0 (0.0)	—
Intrauterine fetal death	0 (0.0)	0 (0.0)	1 (4.0)	0.089
Composite adverse perinatal outcome	31 (16.8) ^a^	19 (27.1) ^a^	19 (76.0) ^b^	<0.001

Data are mean ± standard deviation, median (interquartile range), or *n* (%). Post hoc notation and tests are as in Table 1. Different superscript letters within the same row indicate significant pairwise differences after multiplicity adjustment (adjusted *p* < 0.05); values sharing a letter do not differ significantly. Umbilical artery pH was available in 143, 54, and 19 neonates in the <40, 40–99, and ≥100 µmol/L groups, respectively; Apgar scores were available in 185, 70, and 24 neonates in the same order. NICU length of stay was analyzed among admitted neonates. CAPO, composite adverse perinatal outcome; NICU, neonatal intensive care unit. Pairwise post hoc results were as follows: gestational age at delivery differed significantly across all three pairwise comparisons. For diagnosis-to-delivery interval, the <40 µmol/L group differed from both higher groups, whereas the 40–99 and ≥100 µmol/L groups did not differ. For preterm delivery < 37 weeks, iatrogenic preterm delivery, umbilical artery pH, NICU admission, respiratory distress syndrome, any respiratory support, and CAPO, the ≥100 µmol/L group differed significantly from both lower groups, whereas the <40 and 40–99 µmol/L groups did not differ. For labor induction, only the <40 versus 40–99 µmol/L comparison was significant. For birth weight, 5 min Apgar score, neonatal hypoglycemia, and phototherapy, only the <40 versus ≥100 µmol/L comparison was significant. For meconium-stained amniotic fluid, the <40 µmol/L group differed from both higher categories, whereas the 40–99 and ≥100 µmol/L groups did not differ significantly. Categorical event percentages use the pregnancy denominators shown in the column headings; there were 185, 70, and 24 live births, respectively. The em dash for neonatal death indicates that a comparison was not informative because no events were recorded.

**Table 3:** Diagnostic performance of peak total bile acid thresholds for the composite adverse perinatal outcome.

Overall Discrimination: Apparent AUC 0.700 (95% CI 0.625–0.772), *p* < 0.001.					
Threshold, µmol/L	Sensitivity, % (95% CI)	Specificity, % (95% CI)	LR+	LR−	Youden J
40	55.1 (42.6–67.1)	73.0 (66.5–78.9)	2.04	0.62	0.281
61.3	50.7 (38.4–63.0)	83.9 (78.2–88.6)	3.15	0.59	0.346
100	27.5 (17.5–39.6)	97.2 (93.9–98.9)	9.68	0.75	0.247

Confidence intervals for sensitivity and specificity are exact binomial intervals. The 61.3 µmol/L threshold maximized the Youden index in this cohort and is exploratory. Its performance was evaluated in the derivation cohort; intervals are conditional on the selected threshold and do not account for selection-related optimism. AUC, area under the receiver operating characteristic curve; CI, confidence interval; LR+, positive likelihood ratio; LR−, negative likelihood ratio.

**Table 4:** Univariable and multivariable logistic regression for the composite adverse perinatal outcome.

Variable	Univariable OR (95% CI)	*p* Value	Adjusted OR (95% CI)	*p* Value
Maternal age, per year	1.00 (0.94–1.06)	0.989	—	—
Body mass index, per kg/m^2^	0.98 (0.92–1.04)	0.552	—	—
Nulliparity	0.88 (0.51–1.54)	0.655	—	—
Assisted conception	4.04 (1.05–15.51)	0.042	4.05 (0.88–18.69)	0.073
Smoking	2.53 (0.91–7.08)	0.076	1.98 (0.61–6.44)	0.254
Previous cesarean delivery	1.15 (0.59–2.24)	0.678	—	—
Gestational diabetes mellitus	0.70 (0.23–2.16)	0.538	—	—
Hypertensive disorder	2.58 (1.08–6.19)	0.033	3.27 (1.30–8.23)	0.012
FGR or SGA	1.42 (0.48–4.24)	0.529	—	—
Gestational age at diagnosis, per week	0.72 (0.61–0.86)	<0.001	0.86 (0.71–1.05)	0.130
Peak TBA, per 10 µmol/L	1.23 (1.14–1.33)	<0.001	1.20 (1.11–1.31)	<0.001
AST, per 10 U/L	1.24 (1.06–1.44)	0.006	—	—
ALT, per 10 U/L	1.12 (1.01–1.23)	0.024	—	—
Total bilirubin, per 0.1 mg/dL	1.22 (1.09–1.37)	<0.001	—	—
UDCA use	1.57 (0.57–4.29)	0.383	—	—
Antenatal corticosteroid exposure	14.49 (7.06–29.72)	<0.001	—	—

Variables with univariable *p* < 0.20 were considered for multivariable analysis. AST, ALT, and total bilirubin were not entered with peak TBA because of collinearity; antenatal corticosteroid exposure was excluded because it followed the decision to anticipate early delivery. The final model included assisted conception, smoking, hypertensive disorder, gestational age at diagnosis, and peak TBA. ALT, alanine aminotransferase; AST, aspartate aminotransferase; CI, confidence interval; FGR, fetal growth restriction; OR, odds ratio; SGA, small for gestational age; TBA, total bile acid; UDCA, ursodeoxycholic acid. Assisted conception, smoking, and hypertensive disorder were modeled as binary variables; gestational age at diagnosis and peak TBA were continuous. UDCA use was evaluated in univariable analysis (*p* = 0.383) but was not retained. Gestational age at delivery, UDCA dose, and UDCA initiation timing were not included. An em dash denotes a variable not entered in the adjusted model.

## Data Availability

Patient-level data are not publicly available because of ethical and confidentiality restrictions. Aggregate results are provided in the article and Supplementary Table S1. Access to the underlying hospital records is subject to institutional permission and approval by the relevant ethics committee.

## Supplementary Materials

The following supporting information can be downloaded at https://www.mdpi.com/article/10.3390/jcm15207837/s1, Table S1: Record-based descriptions, individual frequencies, and exact 95% confidence intervals for CAPO components and the composite outcome.

## Abbreviations

The following abbreviations are used in this manuscript:

ALT	Alanine aminotransferase
AST	Aspartate aminotransferase
AUC	Area under the receiver operating characteristic curve
BMI	Body mass index
CAPO	Composite adverse perinatal outcome
CI	Confidence interval
FGR	Fetal growth restriction
GGT	Gamma-glutamyl transferase
ICP	Intrahepatic cholestasis of pregnancy
INR	International normalized ratio
IQR	Interquartile range
LR+	Positive likelihood ratio
LR−	Negative likelihood ratio
NICU	Neonatal intensive care unit
OR	Odds ratio
ROC	Receiver operating characteristic
SD	Standard deviation
SGA	Small for gestational age
TBA	Total bile acid
UDCA	Ursodeoxycholic acid
