# Supplementary material for: Peak Total Bile Acids and Adverse Perinatal Outcomes in Intrahepatic Cholestasis of Pregnancy: A Retrospective Cohort Study with Threshold Analysis
Source: J Clin Med. 2026 Oct 11;15(20):7837. doi: 10.3390/jcm15207837 (PMC13704974; doi:10.3390/jcm15207837)
Supplement: Supplementary file 1 [file jcm-15-07837-s001.zip › jcm-4552286-supplementary.pdf]

Supplementary Material

Peak Total Bile Acids and Adverse Perinatal Outcomes in Intrahepatic Cholestasis of Pregnancy: A Retrospective Cohort Study with Threshold Analysis

Journal of Clinical Medicine

Table S1. Record-based descriptions, individual frequencies, and exact 95% confidence intervals for CAPO components and the composite outcome.

Outcome	Record-based description	Events, *n* (%)	Exact 95% CI, %
Meconium-stained amniotic fluid	Documented meconium in the amniotic fluid.	27 (9.6)	6.45–13.72
Fetal distress or nonreassuring fetal status	Recorded obstetric diagnosis of fetal distress or nonreassuring fetal status.	24 (8.6)	5.57–12.49
NICU admission	Recorded admission to a neonatal intensive care unit.	33 (11.8)	8.25–16.15
Respiratory distress syndrome	Recorded neonatal diagnosis of respiratory distress syndrome; respiratory support alone was not treated as this diagnosis.	4 (1.4)	0.39–3.62
Transient tachypnea of the newborn	Recorded neonatal diagnosis of transient tachypnea; respiratory support alone was not treated as this diagnosis.	6 (2.1)	0.79–4.61
Neonatal death	Recorded death after live birth in the available neonatal outcome records; no uniformly verified 28-day follow-up is implied.	0 (0.0)	0.00–1.31
Intrauterine fetal death	Fetal death after ICP diagnosis and before birth.	1 (0.4)	0.01–1.97
CAPO	At least one of the seven components above. Each pregnancy contributes once, regardless of the number of components; preterm delivery is excluded.	69 (24.6)	19.71–30.12

All percentages and confidence intervals use the full pregnancy cohort as the denominator (N = 280), including the pregnancy ending in intrauterine fetal death, consistent with Table 2. There were 279 live births. Confidence intervals are exact two-sided Clopper–Pearson binomial intervals.

Components overlap and their counts must not be added to estimate CAPO. The composite occurred in 31/185, 19/70, and 19/25 pregnancies in the <40, 40–99, and ≥100 µmol/L peak bile acid categories, respectively. The composite is not severity-weighted and is influenced by clinical management.

Events reflect the recorded clinical diagnoses. Detailed cardiotocographic, radiographic, and neonatal admission criteria were unavailable for standardized re-adjudication. Complete postdischarge mortality ascertainment could not be established.

CAPO, composite adverse perinatal outcome; CI, confidence interval; ICP, intrahepatic cholestasis of pregnancy; NICU, neonatal intensive care unit.
